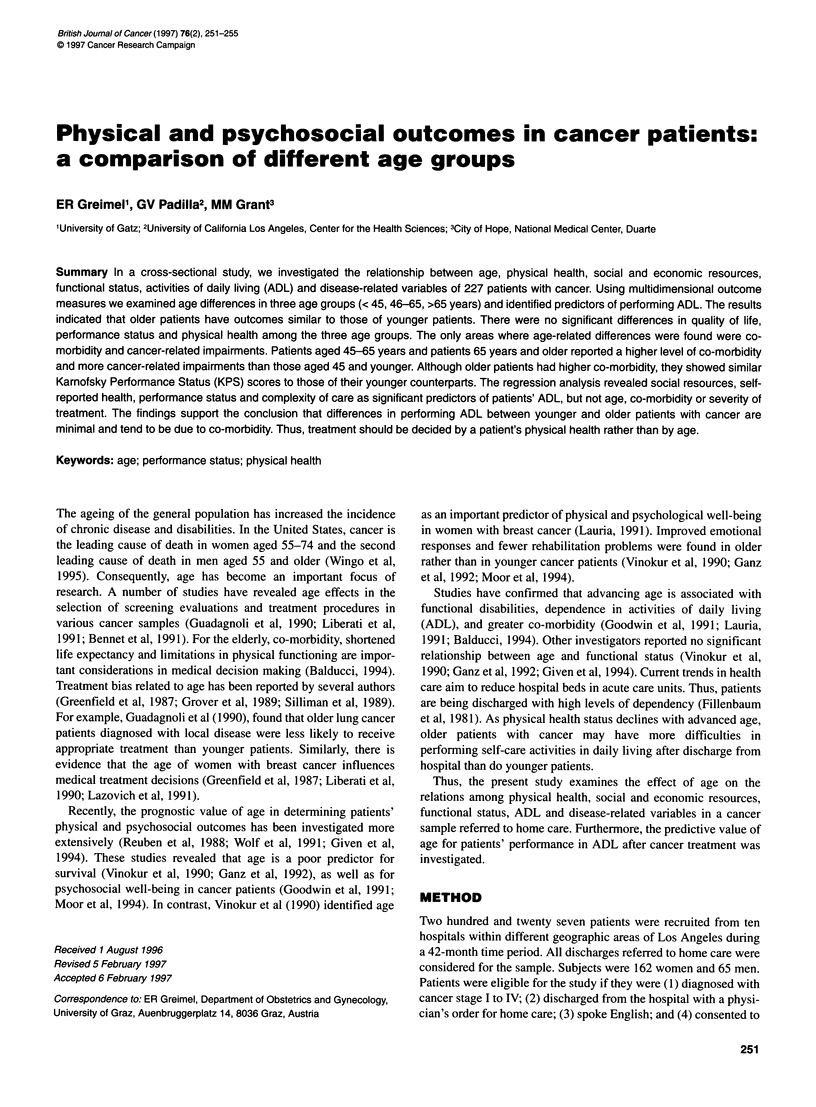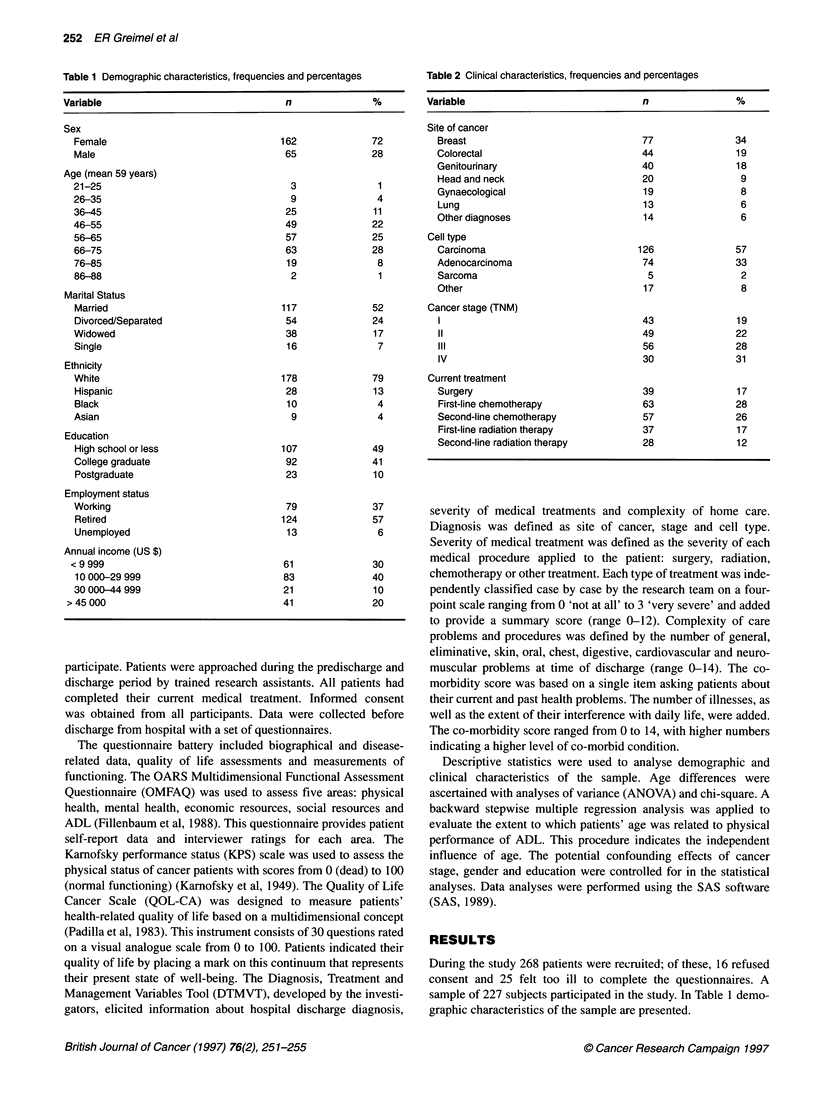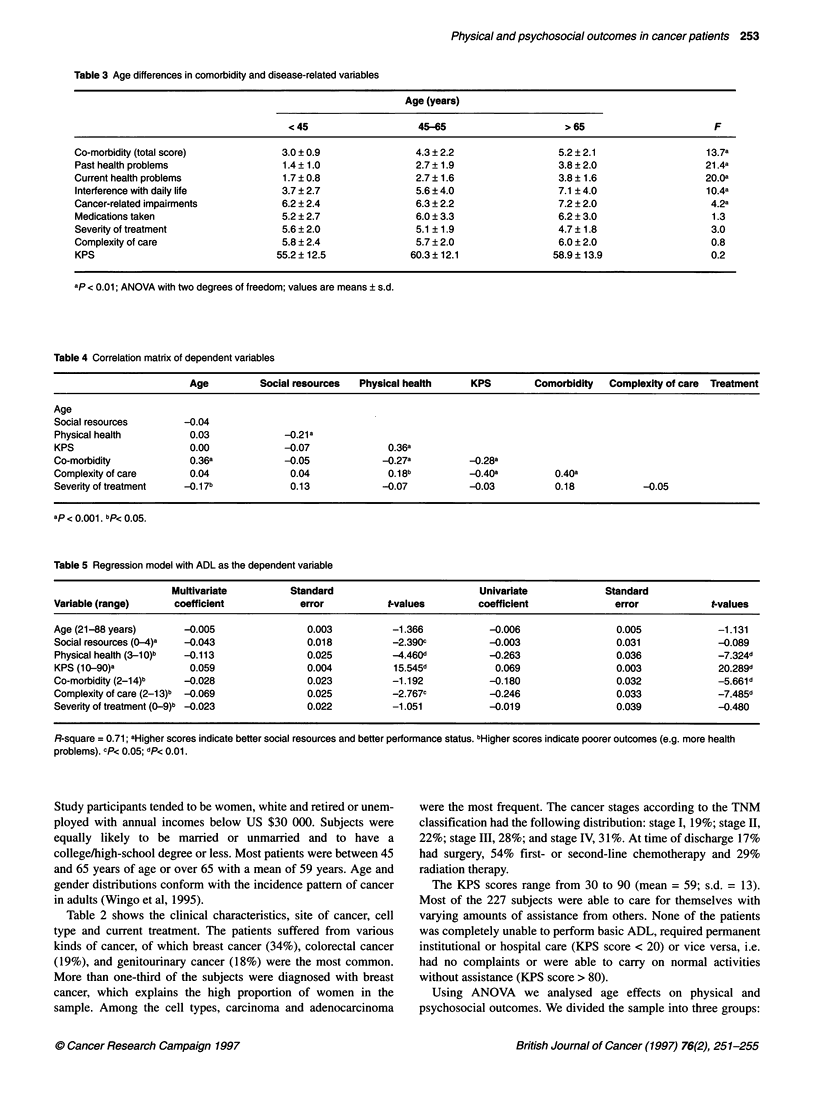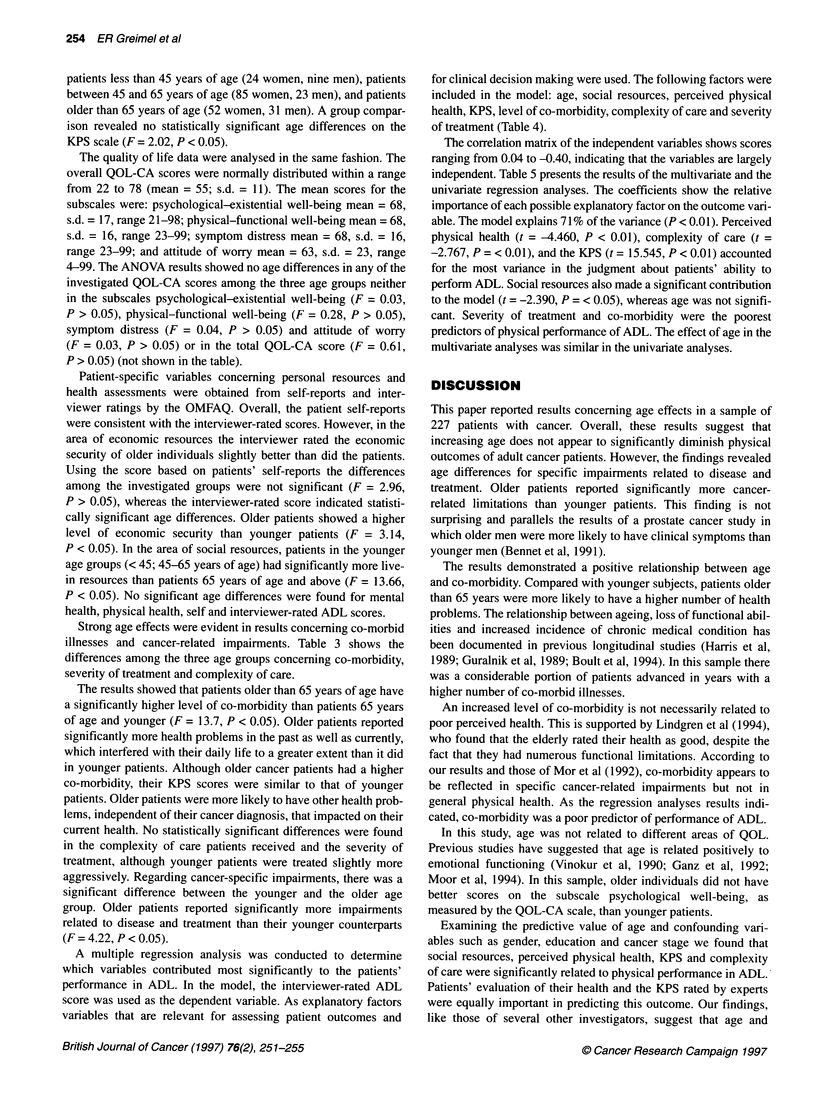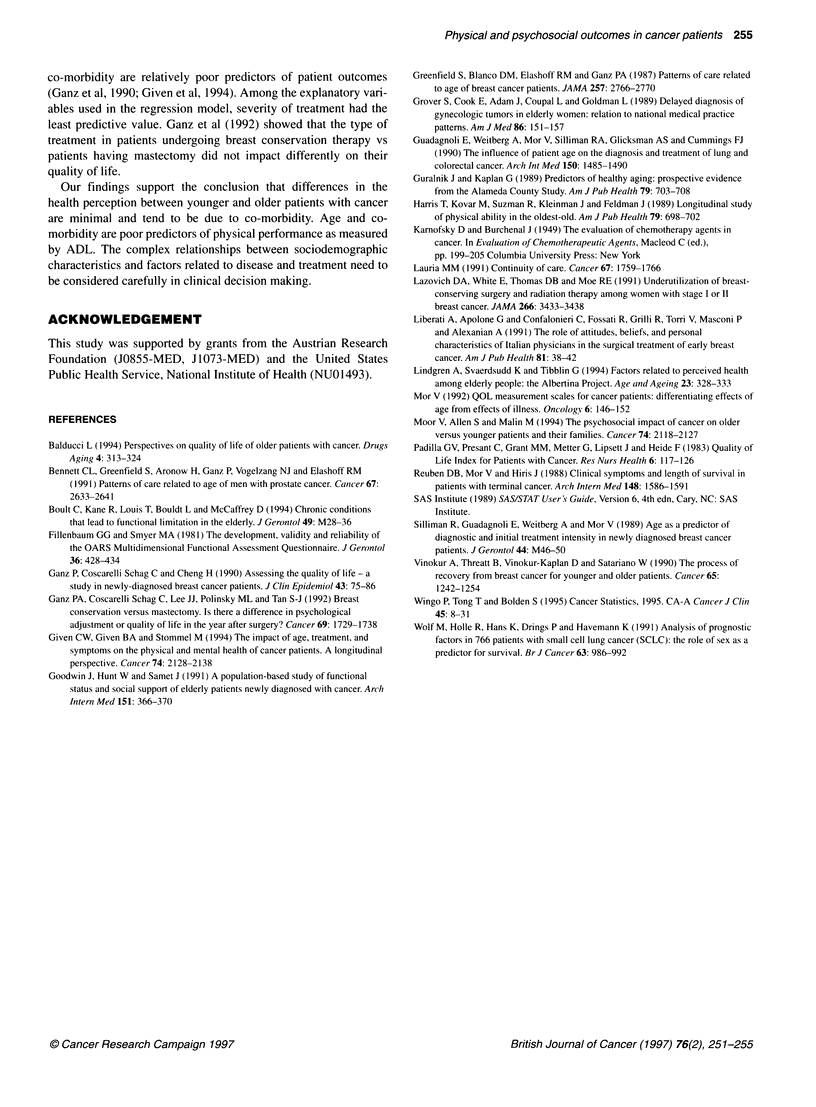# Physical and psychosocial outcomes in cancer patients: a comparison of different age groups.

**DOI:** 10.1038/bjc.1997.370

**Published:** 1997

**Authors:** E. R. Greimel, G. V. Padilla, M. M. Grant

**Affiliations:** University of Gatz, Austria.

## Abstract

In a cross-sectional study, we investigated the relationship between age, physical health, social and economic resources, functional status, activities of daily living (ADL) and disease-related variables of 227 patients with cancer. Using multidimensional outcome measures we examined age differences in three age groups (< 45, 46-65, > 65 years) and identified predictors of performing ADL. The results indicated that older patients have outcomes similar to those of younger patients. There were no significant differences in quality of life, performance status and physical health among the three age groups. The only areas where age-related differences were found were co-morbidity and cancer-related impairments. Patients aged 45-65 years and patients 65 years and older reported a higher level of co-morbidity and more cancer-related impairments than those aged 45 and younger. Although older patients had higher co-morbidity, they showed similar Karnofsky Performance Status (KPS) scores to those of their younger counterparts. The regression analysis revealed social resources, self-reported health, performance status and complexity of care as significant predictors of patients' ADL, but not age, co-morbidity or severity of treatment. The findings support the conclusion that differences in performing ADL between younger and older patients with cancer are minimal and tend to be due to co-morbidity. Thus, treatment should be decided by a patient's physical health rather than by age.


British Journal of Cancer (1997) 76(2), 251–255
© 1997 Cancer Research Campaign

### Physical and psychosocial outcomes in cancer patients: a comparison of different age groups

**ER Greimel[1], GV Padilla[2], MM Grant[3]**

[1]University of Gatz; [2]University of California Los Angeles, Center for the Health Sciences; [3]City of Hope, National Medical Center, Duarte

**Summary** In a cross-sectional study, we investigated the relationship between age, physical health, social and economic resources, functional status, activities of daily living (ADL) and disease-related variables of 227 patients with cancer. Using multidimensional outcome measures we examined age differences in three age groups (< 45, 46–65, >65 years) and identified predictors of performing ADL. The results indicated that older patients have outcomes similar to those of younger patients. There were no significant differences in quality of life, performance status and physical health among the three age groups. The only areas where age-related differences were found were co-morbidity and cancer-related impairments. Patients aged 45–65 years and patients 65 years and older reported a higher level of co-morbidity and more cancer-related impairments than those aged 45 and younger. Although older patients had higher co-morbidity, they showed similar Karnofsky Performance Status (KPS) scores to those of their younger counterparts. The regression analysis revealed social resources, self-reported health, performance status and complexity of care as significant predictors of patients' ADL, but not age, co-morbidity or severity of treatment. The findings support the conclusion that differences in performing ADL between younger and older patients with cancer are minimal and tend to be due to co-morbidity. Thus, treatment should be decided by a patient's physical health rather than by age.

**Keywords:** age; performance status; physical health

The ageing of the general population has increased the incidence of chronic disease and disabilities. In the United States, cancer is the leading cause of death in women aged 55–74 and the second leading cause of death in men aged 55 and older (Wingo et al, 1995). Consequently, age has become an important focus of research. A number of studies have revealed age effects in the selection of screening evaluations and treatment procedures in various cancer samples (Guadagnoli et al, 1990; Liberati et al, 1991; Bennet et al, 1991). For the elderly, co-morbidity, shortened life expectancy and limitations in physical functioning are important considerations in medical decision making (Balducci, 1994). Treatment bias related to age has been reported by several authors (Greenfield et al, 1987; Grover et al, 1989; Silliman et al, 1989). For example, Guadagnoli et al (1990), found that older lung cancer patients diagnosed with local disease were less likely to receive appropriate treatment than younger patients. Similarly, there is evidence that the age of women with breast cancer influences medical treatment decisions (Greenfield et al, 1987; Liberati et al, 1990; Lazovich et al, 1991).

Recently, the prognostic value of age in determining patients' physical and psychosocial outcomes has been investigated more extensively (Reuben et al, 1988; Wolf et al, 1991; Given et al, 1994). These studies revealed that age is a poor predictor for survival (Vinokur et al, 1990; Ganz et al, 1992), as well as for psychosocial well-being in cancer patients (Goodwin et al, 1991; Moor et al, 1994). In contrast, Vinokur et al (1990) identified age as an important predictor of physical and psychological well-being in women with breast cancer (Lauria, 1991). Improved emotional responses and fewer rehabilitation problems were found in older rather than in younger cancer patients (Vinokur et al, 1990; Ganz et al, 1992; Moor et al, 1994).

Studies have confirmed that advancing age is associated with functional disabilities, dependence in activities of daily living (ADL), and greater co-morbidity (Goodwin et al, 1991; Lauria, 1991; Balducci, 1994). Other investigators reported no significant relationship between age and functional status (Vinokur et al, 1990; Ganz et al, 1992; Given et al, 1994). Current trends in health care aim to reduce hospital beds in acute care units. Thus, patients are being discharged with high levels of dependency (Fillenbaum et al, 1981). As physical health status declines with advanced age, older patients with cancer may have more difficulties in performing self-care activities in daily living after discharge from hospital than do younger patients.

Thus, the present study examines the effect of age on the relations among physical health, social and economic resources, functional status, ADL and disease-related variables in a cancer sample referred to home care. Furthermore, the predictive value of age for patients' performance in ADL after cancer treatment was investigated.

### METHOD

Two hundred and twenty seven patients were recruited from ten hospitals within different geographic areas of Los Angeles during a 42-month time period. All discharges referred to home care were considered for the sample. Subjects were 162 women and 65 men. Patients were eligible for the study if they were (1) diagnosed with cancer stage I to IV; (2) discharged from the hospital with a physician's order for home care; (3) spoke English; and (4) consented to

*Received 1 August 1996*
*Revised 5 February 1997*
*Accepted 6 February 1997*

*Correspondence to:* ER Greimel, Department of Obstetrics and Gynecology, University of Graz, Auenbruggerplatz 14, 8036 Graz, Austria

Table 1 Demographic characteristics, frequencies and percentages

| Variable | n | % |
|---|---|---|
| Sex | | |
| Female | 162 | 72 |
| Male | 65 | 28 |
| Age (mean 59 years) | | |
| 21–25 | 3 | 1 |
| 26–35 | 9 | 4 |
| 36–45 | 25 | 11 |
| 46–55 | 49 | 22 |
| 56–65 | 57 | 25 |
| 66–75 | 63 | 28 |
| 76–85 | 19 | 8 |
| 86–88 | 2 | 1 |
| Marital Status | | |
| Married | 117 | 52 |
| Divorced/Separated | 54 | 24 |
| Widowed | 38 | 17 |
| Single | 16 | 7 |
| Ethnicity | | |
| White | 178 | 79 |
| Hispanic | 28 | 13 |
| Black | 10 | 4 |
| Asian | 9 | 4 |
| Education | | |
| High school or less | 107 | 49 |
| College graduate | 92 | 41 |
| Postgraduate | 23 | 10 |
| Employment status | | |
| Working | 79 | 37 |
| Retired | 124 | 57 |
| Unemployed | 13 | 6 |
| Annual income (US $) | | |
| < 9 999 | 61 | 30 |
| 10 000–29 999 | 83 | 40 |
| 30 000–44 999 | 21 | 10 |
| > 45 000 | 41 | 20 |

Table 2 Clinical characteristics, frequencies and percentages

| Variable | n | % |
|---|---|---|
| Site of cancer | | |
| Breast | 77 | 34 |
| Colorectal | 44 | 19 |
| Genitourinary | 40 | 18 |
| Head and neck | 20 | 9 |
| Gynaecological | 19 | 8 |
| Lung | 13 | 6 |
| Other diagnoses | 14 | 6 |
| Cell type | | |
| Carcinoma | 126 | 57 |
| Adenocarcinoma | 74 | 33 |
| Sarcoma | 5 | 2 |
| Other | 17 | 8 |
| Cancer stage (TNM) | | |
| I | 43 | 19 |
| II | 49 | 22 |
| III | 56 | 28 |
| IV | 30 | 31 |
| Current treatment | | |
| Surgery | 39 | 17 |
| First-line chemotherapy | 63 | 28 |
| Second-line chemotherapy | 57 | 26 |
| First-line radiation therapy | 37 | 17 |
| Second-line radiation therapy | 28 | 12 |

participate. Patients were approached during the predischarge and discharge period by trained research assistants. All patients had completed their current medical treatment. Informed consent was obtained from all participants. Data were collected before discharge from hospital with a set of questionnaires.

The questionnaire battery included biographical and disease-related data, quality of life assessments and measurements of functioning. The OARS Multidimensional Functional Assessment Questionnaire (OMFAQ) was used to assess five areas: physical health, mental health, economic resources, social resources and ADL (Fillenbaum et al, 1988). This questionnaire provides patient self-report data and interviewer ratings for each area. The Karnofsky performance status (KPS) scale was used to assess the physical status of cancer patients with scores from 0 (dead) to 100 (normal functioning) (Karnofsky et al, 1949). The Quality of Life Cancer Scale (QOL-CA) was designed to measure patients' health-related quality of life based on a multidimensional concept (Padilla et al, 1983). This instrument consists of 30 questions rated on a visual analogue scale from 0 to 100. Patients indicated their quality of life by placing a mark on this continuum that represents their present state of well-being. The Diagnosis, Treatment and Management Variables Tool (DTMVT), developed by the investigators, elicited information about hospital discharge diagnosis, severity of medical treatments and complexity of home care. Diagnosis was defined as site of cancer, stage and cell type. Severity of medical treatment was defined as the severity of each medical procedure applied to the patient: surgery, radiation, chemotherapy or other treatment. Each type of treatment was independently classified case by case by the research team on a four-point scale ranging from 0 'not at all' to 3 'very severe' and added to provide a summary score (range 0–12). Complexity of care problems and procedures was defined by the number of general, eliminative, skin, oral, chest, digestive, cardiovascular and neuromuscular problems at time of discharge (range 0–14). The co-morbidity score was based on a single item asking patients about their current and past health problems. The number of illnesses, as well as the extent of their interference with daily life, were added. The co-morbidity score ranged from 0 to 14, with higher numbers indicating a higher level of co-morbid condition.

Descriptive statistics were used to analyse demographic and clinical characteristics of the sample. Age differences were ascertained with analyses of variance (ANOVA) and chi-square. A backward stepwise multiple regression analysis was applied to evaluate the extent to which patients' age was related to physical performance of ADL. This procedure indicates the independent influence of age. The potential confounding effects of cancer stage, gender and education were controlled for in the statistical analyses. Data analyses were performed using the SAS software (SAS, 1989).

### RESULTS

During the study 268 patients were recruited; of these, 16 refused consent and 25 felt too ill to complete the questionnaires. A sample of 227 subjects participated in the study. In Table 1 demographic characteristics of the sample are presented.

© Cancer Research Campaign 1997

**Table 3** Age differences in comorbidity and disease-related variables

| | Age (years) | | | |
|---|---|---|---|---|
| | < 45 | 45–65 | > 65 | *F* |
| Co-morbidity (total score) | 3.0 ± 0.9 | 4.3 ± 2.2 | 5.2 ± 2.1 | 13.7[a] |
| Past health problems | 1.4 ± 1.0 | 2.7 ± 1.9 | 3.8 ± 2.0 | 21.4[a] |
| Current health problems | 1.7 ± 0.8 | 2.7 ± 1.6 | 3.8 ± 1.6 | 20.0[a] |
| Interference with daily life | 3.7 ± 2.7 | 5.6 ± 4.0 | 7.1 ± 4.0 | 10.4[a] |
| Cancer-related impairments | 6.2 ± 2.4 | 6.3 ± 2.2 | 7.2 ± 2.0 | 4.2[a] |
| Medications taken | 5.2 ± 2.7 | 6.0 ± 3.3 | 6.2 ± 3.0 | 1.3 |
| Severity of treatment | 5.6 ± 2.0 | 5.1 ± 1.9 | 4.7 ± 1.8 | 3.0 |
| Complexity of care | 5.8 ± 2.4 | 5.7 ± 2.0 | 6.0 ± 2.0 | 0.8 |
| KPS | 55.2 ± 12.5 | 60.3 ± 12.1 | 58.9 ± 13.9 | 0.2 |

[a]$P < 0.01$; ANOVA with two degrees of freedom; values are means ± s.d.

**Table 4** Correlation matrix of dependent variables

| | Age | Social resources | Physical health | KPS | Comorbidity | Complexity of care | Treatment |
|---|---|---|---|---|---|---|---|
| Age | | | | | | | |
| Social resources | –0.04 | | | | | | |
| Physical health | 0.03 | –0.21[a] | | | | | |
| KPS | 0.00 | –0.07 | 0.36[a] | | | | |
| Co-morbidity | 0.36[a] | –0.05 | –0.27[a] | –0.28[a] | | | |
| Complexity of care | 0.04 | 0.04 | 0.18[b] | –0.40[a] | 0.40[a] | | |
| Severity of treatment | –0.17[b] | 0.13 | –0.07 | –0.03 | 0.18 | –0.05 | |

[a]$P < 0.001$. [b]$P < 0.05$.

**Table 5** Regression model with ADL as the dependent variable

| Variable (range) | Multivariate coefficient | Standard error | *t*-values | Univariate coefficient | Standard error | *t*-values |
|---|---|---|---|---|---|---|
| Age (21–88 years) | –0.005 | 0.003 | –1.366 | –0.006 | 0.005 | –1.131 |
| Social resources (0–4)[a] | –0.043 | 0.018 | –2.390[c] | –0.003 | 0.031 | –0.089 |
| Physical health (3–10)[b] | –0.113 | 0.025 | –4.460[d] | –0.263 | 0.036 | –7.324[d] |
| KPS (10–90)[a] | 0.059 | 0.004 | 15.545[d] | 0.069 | 0.003 | 20.289[d] |
| Co-morbidity (2–14)[b] | –0.028 | 0.023 | –1.192 | –0.180 | 0.032 | –5.661[d] |
| Complexity of care (2–13)[b] | –0.069 | 0.025 | –2.767[c] | –0.246 | 0.033 | –7.485[d] |
| Severity of treatment (0–9)[b] | –0.023 | 0.022 | –1.051 | –0.019 | 0.039 | –0.480 |

$R$-square = 0.71; [a]Higher scores indicate better social resources and better performance status. [b]Higher scores indicate poorer outcomes (e.g. more health problems). [c]$P < 0.05$; [d]$P < 0.01$.

Study participants tended to be women, white and retired or unemployed with annual incomes below US $30 000. Subjects were equally likely to be married or unmarried and to have a college/high-school degree or less. Most patients were between 45 and 65 years of age or over 65 with a mean of 59 years. Age and gender distributions conform with the incidence pattern of cancer in adults (Wingo et al, 1995).

Table 2 shows the clinical characteristics, site of cancer, cell type and current treatment. The patients suffered from various kinds of cancer, of which breast cancer (34%), colorectal cancer (19%), and genitourinary cancer (18%) were the most common. More than one-third of the subjects were diagnosed with breast cancer, which explains the high proportion of women in the sample. Among the cell types, carcinoma and adenocarcinoma were the most frequent. The cancer stages according to the TNM classification had the following distribution: stage I, 19%; stage II, 22%; stage III, 28%; and stage IV, 31%. At time of discharge 17% had surgery, 54% first- or second-line chemotherapy and 29% radiation therapy.

The KPS scores range from 30 to 90 (mean = 59; s.d. = 13). Most of the 227 subjects were able to care for themselves with varying amounts of assistance from others. None of the patients was completely unable to perform basic ADL, required permanent institutional or hospital care (KPS score < 20) or vice versa, i.e. had no complaints or were able to carry on normal activities without assistance (KPS score > 80).

Using ANOVA we analysed age effects on physical and psychosocial outcomes. We divided the sample into three groups:

patients less than 45 years of age (24 women, nine men), patients between 45 and 65 years of age (85 women, 23 men), and patients older than 65 years of age (52 women, 31 men). A group comparison revealed no statistically significant age differences on the KPS scale ($F = 2.02$, $P < 0.05$).

The quality of life data were analysed in the same fashion. The overall QOL-CA scores were normally distributed within a range from 22 to 78 (mean = 55; s.d. = 11). The mean scores for the subscales were: psychological–existential well-being mean = 68, s.d. = 17, range 21–98; physical–functional well-being mean = 68, s.d. = 16, range 23–99; symptom distress mean = 68, s.d. = 16, range 23–99; and attitude of worry mean = 63, s.d. = 23, range 4–99. The ANOVA results showed no age differences in any of the investigated QOL-CA scores among the three age groups neither in the subscales psychological–existential well-being ($F = 0.03$, $P > 0.05$), physical–functional well-being ($F = 0.28$, $P > 0.05$), symptom distress ($F = 0.04$, $P > 0.05$) and attitude of worry ($F = 0.03$, $P > 0.05$) or in the total QOL-CA score ($F = 0.61$, $P > 0.05$) (not shown in the table).

Patient-specific variables concerning personal resources and health assessments were obtained from self-reports and interviewer ratings by the OMFAQ. Overall, the patient self-reports were consistent with the interviewer-rated scores. However, in the area of economic resources the interviewer rated the economic security of older individuals slightly better than did the patients. Using the score based on patients' self-reports the differences among the investigated groups were not significant ($F = 2.96$, $P > 0.05$), whereas the interviewer-rated score indicated statistically significant age differences. Older patients showed a higher level of economic security than younger patients ($F = 3.14$, $P < 0.05$). In the area of social resources, patients in the younger age groups (< 45; 45–65 years of age) had significantly more live-in resources than patients 65 years of age and above ($F = 13.66$, $P < 0.05$). No significant age differences were found for mental health, physical health, self and interviewer-rated ADL scores.

Strong age effects were evident in results concerning co-morbid illnesses and cancer-related impairments. Table 3 shows the differences among the three age groups concerning co-morbidity, severity of treatment and complexity of care.

The results showed that patients older than 65 years of age have a significantly higher level of co-morbidity than patients 65 years of age and younger ($F = 13.7$, $P < 0.05$). Older patients reported significantly more health problems in the past as well as currently, which interfered with their daily life to a greater extent than it did in younger patients. Although older cancer patients had a higher co-morbidity, their KPS scores were similar to that of younger patients. Older patients were more likely to have other health problems, independent of their cancer diagnosis, that impacted on their current health. No statistically significant differences were found in the complexity of care patients received and the severity of treatment, although younger patients were treated slightly more aggressively. Regarding cancer-specific impairments, there was a significant difference between the younger and the older age group. Older patients reported significantly more impairments related to disease and treatment than their younger counterparts ($F = 4.22$, $P < 0.05$).

A multiple regression analysis was conducted to determine which variables contributed most significantly to the patients' performance in ADL. In the model, the interviewer-rated ADL score was used as the dependent variable. As explanatory factors variables that are relevant for assessing patient outcomes and for clinical decision making were used. The following factors were included in the model: age, social resources, perceived physical health, KPS, level of co-morbidity, complexity of care and severity of treatment (Table 4).

The correlation matrix of the independent variables shows scores ranging from 0.04 to –0.40, indicating that the variables are largely independent. Table 5 presents the results of the multivariate and the univariate regression analyses. The coefficients show the relative importance of each possible explanatory factor on the outcome variable. The model explains 71% of the variance ($P < 0.01$). Perceived physical health ($t = -4.460$, $P < 0.01$), complexity of care ($t = -2.767$, $P = < 0.01$), and the KPS ($t = 15.545$, $P < 0.01$) accounted for the most variance in the judgment about patients' ability to perform ADL. Social resources also made a significant contribution to the model ($t = -2.390$, $P = < 0.05$), whereas age was not significant. Severity of treatment and co-morbidity were the poorest predictors of physical performance of ADL. The effect of age in the multivariate analyses was similar in the univariate analyses.

### DISCUSSION

This paper reported results concerning age effects in a sample of 227 patients with cancer. Overall, these results suggest that increasing age does not appear to significantly diminish physical outcomes of adult cancer patients. However, the findings revealed age differences for specific impairments related to disease and treatment. Older patients reported significantly more cancer-related limitations than younger patients. This finding is not surprising and parallels the results of a prostate cancer study in which older men were more likely to have clinical symptoms than younger men (Bennet et al, 1991).

The results demonstrated a positive relationship between age and co-morbidity. Compared with younger subjects, patients older than 65 years were more likely to have a higher number of health problems. The relationship between ageing, loss of functional abilities and increased incidence of chronic medical condition has been documented in previous longitudinal studies (Harris et al, 1989; Guralnik et al, 1989; Boult et al, 1994). In this sample there was a considerable portion of patients advanced in years with a higher number of co-morbid illnesses.

An increased level of co-morbidity is not necessarily related to poor perceived health. This is supported by Lindgren et al (1994), who found that the elderly rated their health as good, despite the fact that they had numerous functional limitations. According to our results and those of Mor et al (1992), co-morbidity appears to be reflected in specific cancer-related impairments but not in general physical health. As the regression analyses results indicated, co-morbidity was a poor predictor of performance of ADL.

In this study, age was not related to different areas of QOL. Previous studies have suggested that age is related positively to emotional functioning (Vinokur et al, 1990; Ganz et al, 1992; Moor et al, 1994). In this sample, older individuals did not have better scores on the subscale psychological well-being, as measured by the QOL-CA scale, than younger patients.

Examining the predictive value of age and confounding variables such as gender, education and cancer stage we found that social resources, perceived physical health, KPS and complexity of care were significantly related to physical performance in ADL. Patients' evaluation of their health and the KPS rated by experts were equally important in predicting this outcome. Our findings, like those of several other investigators, suggest that age and

© Cancer Research Campaign 1997

co-morbidity are relatively poor predictors of patient outcomes (Ganz et al, 1990; Given et al, 1994). Among the explanatory variables used in the regression model, severity of treatment had the least predictive value. Ganz et al (1992) showed that the type of treatment in patients undergoing breast conservation therapy vs patients having mastectomy did not impact differently on their quality of life.

Our findings support the conclusion that differences in the health perception between younger and older patients with cancer are minimal and tend to be due to co-morbidity. Age and co-morbidity are poor predictors of physical performance as measured by ADL. The complex relationships between sociodemographic characteristics and factors related to disease and treatment need to be considered carefully in clinical decision making.

### ACKNOWLEDGEMENT

This study was supported by grants from the Austrian Research Foundation (J0855-MED, J1073-MED) and the United States Public Health Service, National Institute of Health (NU01493).

### REFERENCES

Balducci L (1994) Perspectives on quality of life of older patients with cancer. *Drugs Aging* **4**: 313–324

Bennett CL, Greenfield S, Aronow H, Ganz P, Vogelzang NJ and Elashoff RM (1991) Patterns of care related to age of men with prostate cancer. *Cancer* **67**: 2633–2641

Boult C, Kane R, Louis T, Bouldt L and McCaffrey D (1994) Chronic conditions that lead to functional limitation in the elderly. *J Gerontol* **49**: M28–36

Fillenbaum GG and Smyer MA (1981) The development, validity and reliability of the OARS Multidimensional Functional Assessment Questionnaire. *J Gerontol* **36**: 428–434

Ganz P, Coscarelli Schag C and Cheng H (1990) Assessing the quality of life – a study in newly-diagnosed breast cancer patients. *J Clin Epidemiol* **43**: 75–86

Ganz PA, Coscarelli Schag C, Lee JJ, Polinsky ML and Tan S-J (1992) Breast conservation versus mastectomy. Is there a difference in psychological adjustment or quality of life in the year after surgery? *Cancer* **69**: 1729–1738

Given CW, Given BA and Stommel M (1994) The impact of age, treatment, and symptoms on the physical and mental health of cancer patients. A longitudinal perspective. *Cancer* **74**: 2128–2138

Goodwin J, Hunt W and Samet J (1991) A population-based study of functional status and social support of elderly patients newly diagnosed with cancer. *Arch Intern Med* **151**: 366–370

Greenfield S, Blanco DM, Elashoff RM and Ganz PA (1987) Patterns of care related to age of breast cancer patients. *JAMA* **257**: 2766–2770

Grover S, Cook E, Adam J, Coupal L and Goldman L (1989) Delayed diagnosis of gynecologic tumors in elderly women: relation to national medical practice patterns. *Am J Med* **86**: 151–157

Guadagnoli E, Weitberg A, Mor V, Silliman RA, Glicksman AS and Cummings FJ (1990) The influence of patient age on the diagnosis and treatment of lung and colorectal cancer. *Arch Int Med* **150**: 1485–1490

Guralnik J and Kaplan G (1989) Predictors of healthy aging: prospective evidence from the Alameda County Study. *Am J Pub Health* **79**: 703–708

Harris T, Kovar M, Suzman R, Kleinman J and Feldman J (1989) Longitudinal study of physical ability in the oldest-old. *Am J Pub Health* **79**: 698–702

Karnofsky D and Burchenal J (1949) The evaluation of chemotherapy agents in cancer. In *Evaluation of Chemotherapeutic Agents*, Macleod C (ed.), pp. 199–205 Columbia University Press: New York

Lauria MM (1991) Continuity of care. *Cancer* **67**: 1759–1766

Lazovich DA, White E, Thomas DB and Moe RE (1991) Underutilization of breast-conserving surgery and radiation therapy among women with stage I or II breast cancer. *JAMA* **266**: 3433–3438

Liberati A, Apolone G and Confalonieri C, Fossati R, Grilli R, Torri V, Masconi P and Alexanian A (1991) The role of attitudes, beliefs, and personal characteristics of Italian physicians in the surgical treatment of early breast cancer. *Am J Pub Health* **81**: 38–42

Lindgren A, Svaerdsudd K and Tibblin G (1994) Factors related to perceived health among elderly people: the Albertina Project. *Age and Ageing* **23**: 328–333

Mor V (1992) QOL measurement scales for cancer patients: differentiating effects of age from effects of illness. *Oncology* **6**: 146–152

Moor V, Allen S and Malin M (1994) The psychosocial impact of cancer on older versus younger patients and their families. *Cancer* **74**: 2118–2127

Padilla GV, Presant C, Grant MM, Metter G, Lipsett J and Heide F (1983) Quality of Life Index for Patients with Cancer. *Res Nurs Health* **6**: 117–126

Reuben DB, Mor V and Hiris J (1988) Clinical symptoms and length of survival in patients with terminal cancer. *Arch Intern Med* **148**: 1586–1591

SAS Institute (1989) *SAS/STAT User's Guide*, Version 6, 4th edn, Cary, NC: SAS Institute.

Silliman R, Guadagnoli E, Weitberg A and Mor V (1989) Age as a predictor of diagnostic and initial treatment intensity in newly diagnosed breast cancer patients. *J Gerontol* **44**: M46–50

Vinokur A, Threatt B, Vinokur-Kaplan D and Satariano W (1990) The process of recovery from breast cancer for younger and older patients. *Cancer* **65**: 1242–1254

Wingo P, Tong T and Bolden S (1995) Cancer Statistics, 1995. CA-A *Cancer J Clin* **45**: 8–31

Wolf M, Holle R, Hans K, Drings P and Havemann K (1991) Analysis of prognostic factors in 766 patients with small cell lung cancer (SCLC): the role of sex as a predictor for survival. *Br J Cancer* **63**: 986–992

© Cancer Research Campaign 1997